# Response of bacterial community metabolites to bacterial wilt caused by *Ralstonia solanacearum*: a multi-omics analysis

**DOI:** 10.3389/fpls.2023.1339478

**Published:** 2024-01-22

**Authors:** Chengjian Wei, Jinchang Liang, Rui Wang, Luping Chi, Wenjing Wang, Jun Tan, Heli Shi, Xueru Song, Zhenzhen Cui, Qiang Xie, Dejie Cheng, Xiaoqiang Wang

**Affiliations:** ^1^ College of Agriculture, Guangxi University, Nanning, China; ^2^ Key Laboratory of Tobacco Pest Monitoring & Integrated Management, Tobacco Research Institute of Chinese Academy of Agricultural Sciences, Qingdao, China; ^3^ Enshi Tobacco Science and Technology Center, Enshi, China; ^4^ Engineering Center for Biological Control of Diseases and Pests in Tobacco Industry, Yuxi, China; ^5^ Sichuan Tobacco Science and Technology Center, Chengdu, China

**Keywords:** keystone taxa, metabolites, bacterial wilt, rhizosphere, microbiomes

## Abstract

The soil microbial community plays a critical role in promoting robust plant growth and serves as an effective defence mechanism against root pathogens. Current research has focused on unravelling the compositions and functions of diverse microbial taxa in plant rhizospheres invaded by *Ralstonia solanacearum*, however, the specific mechanisms by which key microbial groups with distinct functions exert their effects remain unclear. In this study, we employed a combination of amplicon sequencing and metabolomics analysis to investigate the principal metabolic mechanisms of key microbial taxa in plant rhizosphere soil. Compared to the healthy tobacco rhizosphere samples, the bacterial diversity and co-occurrence network of the diseased tobacco rhizosphere soil were significantly reduced. Notably, certain genera, including *Gaiella*, *Rhodoplanes*, and MND1 (*Nitrosomonadaceae*), were found to be significantly more abundant in the rhizosphere of healthy plants than in that of diseased plants. Eight environmental factors, including exchangeable magnesium, available phosphorus, and pH, were found to be crucial factors influencing the composition of the microbial community. *Ralstonia* displayed negative correlations with pH, exchangeable magnesium, and cation exchange flux, but showed a positive correlation with available iron. Furthermore, metabolomic analysis revealed that the metabolic pathways related to the synthesis of various antibacterial compounds were significantly enriched in the healthy group. The correlation analysis results indicate that the bacterial genera *Polycyclovorans*, *Lysobacter*, *Pseudomonas*, and *Nitrosospira* may participate in the synthesis of antibacterial compounds. Collectively, our findings contribute to a more in-depth understanding of disease resistance mechanisms within healthy microbial communities and provide a theoretical foundation for the development of targeted strategies using beneficial microorganisms to suppress disease occurrence.

## Introduction

Bacterial wilt is a severely damaging vascular disease with a wide host range, including economically important crops, such as tomato, potato, eggplant, pepper, and tobacco (Hasegawa et al., 2019). The causal pathogen, *Ralstonia solanacearum*, infiltrates solanaceous plants through root wounds, establishes colonies, and eventually enters the xylem, resulting in necrosis and wilting of the infected plants. The resulting damage to crops leads to substantial economic losses annually (Prior et al., 2016). The results of numerous studies have confirmed the essential role of the rhizosphere microbial communities in plant health and growth (Besset-Manzoni et al., 2018; Kashyap et al., 2023). Plants adjust their rhizosphere microbial composition in response to pathogen infections by selectively recruiting a group of beneficial microorganisms that induce disease resistance and promote growth, thereby altering the structure of the microbial community (Berendsen et al., 2018). For example, *Streptomyces* has been found to promote plant growth, which helps to improve microbial community diversity in the rhizosphere. In addition, some bacterial genera such as *Bacillus* and *Pseudomonas* can improve the resistance of plants to diseases (Zheng et al., 2019; Gashaw et al., 2022). In agricultural ecosystems, rhizosphere microbial communities significantly affect the growth, development, and resistance of plants to soil-borne diseases (Mendes et al., 2018; Kashyap et al., 2021). Most studies have focused on the changes in bacterial community composition during pathogen invasion; however, the key bacterial taxon that may participate in the defence against pathogens remains unclear.

The keystone taxa within the rhizosphere microbial community play a crucial role in maintaining soil community structure stability and promoting plant health (Oberholster et al., 2018). For example, key soil microbial groups alter the flow of minerals between crops and soil, thereby improving crop production (Wang et al., 2022b). Certain keystone taxa play dominant roles in denitrification, which is a key driving factor in microbial nitrogen cycling. Additionally, some specific keystone taxa possess unique functions, such as nitrogen metabolism or phosphonate and phosphite metabolism, which are crucial for maintaining the stability of the soil microbial community (Xun et al., 2021). In recent years, network analysis has been widely applied to visualize the interrelationships within microbial communities and identify keystone taxa. Within microbial symbiotic networks, microbial taxa that are highly interconnected with other taxa are often considered keystone species that potentially exert a significant influence on the microbial community structure (Cardona et al., 2016; Herren and McMahon, 2018). Through network analysis, the soil bacterial networks and community structures of plots of healthy and diseased tomato plants were compared and bacterial strains with disease-suppressing activity were successfully identified (Zhang et al., 2020).

The metabolites produced by plants and rhizosphere microorganisms are crucial for regulating plant health (Bi et al., 2021). Plants interact with metabolites produced by their roots and rhizosphere microorganisms, and root exudates play a role in selectively influencing the rhizosphere environment through biological suppression and signaling activities (Pétriacq et al., 2017). For example, *Arabidopsis* plants selectively recruit *Bacillus* spp. by releasing malic acid from the roots, thereby improving disease resistance (Rudrappa et al., 2008). In the case of carnation (*Dianthus caryophyllus*) protection against pathogenic bacteria is a result of increased concentration of flavonol glycosides (Hassan and Mathesius, 2012). Microbial metabolites affect plant nutrient absorption, health, and soil biodiversity. For example, *Streptomyces* AN090126 produces various antibacterial secondary metabolites, including dimethyl sulfide and trimethyl sulfide, and exhibits broad-spectrum antagonistic activity against various plant pathogenic bacteria (Le et al., 2022). The secondary metabolites produced by *Trichoderma* can activate the disease resistance mechanism in plants and prevent pathogen invasion (Manzar et al., 2021; Manzar et al., 2022). *Bacillus cereus* has been shown to regulate salicylic acid and jasmonic acid signaling pathways in plants, promoting the aggregation of beneficial microorganisms in the rhizosphere, and thus controlling the growth of pathogenic bacteria (Yang et al., 2023a). However, relatively little research has been conducted on the metabolomics of plant rhizospheres during bacterial wilt infection. The rhizosphere keystone metabolites and associated metabolic pathways involved in regulating tobacco pathogen infection are not clear, and the potential keystone taxa that drive these essential metabolic functions have also not been identified.

In this study, we compared the community composition and metabolic profiles of the rhizosphere soil between tobacco plants infected with bacterial wilt and healthy plants. Variations in the rhizosphere soil microbial communities of tobacco plants under different health conditions were compared using amplicon sequencing, and the key environmental factors influencing soil microbial communities were identified. In addition, the keystone bacterial taxa in the rhizosphere soil of healthy plants that may contribute to bacterial wilt resistance were identified using co-occurrence network analysis. Furthermore, significantly enriched metabolic pathways in rhizosphere soil samples from healthy plants and their potential functions were investigated, and the relationships between different metabolites and keystone bacterial genera were established. The results of this study not only deepen our understanding of the roles of rhizosphere microbiomes in plant hosts, but also reveal the mechanisms of resisting pathogens of keystone microbes.

## Materials and methods

### Sample collection

Soil samples were collected in the tobacco-producing fields of Xuan’en County (29°59″1.932″N,109°35″2.976″E) in Hubei Province, China, where tobacco has been planted for decades. Bacterial wilt disease occurred beginning from 40 days after transplanting (about mid-June) every year, and the incidence rate of bacterial wilt was even more than 30% after the flowering stage, resulting in a 20-30% reduction in tobacco yield. Samples were collected from tobacco plants infected by the disease and from healthy tobacco plants growing in the adjacent field on July 29, 2022 (80 days after transplanting), thus minimizing any geographical and environmental influences. Six plants (cultivar Yunyan 87) displaying light symptoms (grade 1 infection) (Standard, China, 2008) of tobacco bacterial wilt were selected, along with six healthy plants that exhibited similar growth as healthy controls. Rhizosphere soil was collected according to the method described by Yang et al. with some modifications (Yang et al., 2017). Specifically, the selected tobacco plants were carefully uprooted and the loosely adhered soil was shaken off. Fine roots collected from the same plant were combined as one sample and the soil on the surface (within approximately 1-2 mm) of the fine roots was brushed using a soft brush, which was defined as rhizosphere soil. Twelve rhizosphere soil samples were collected and stored at -80°C until used in the experiments.

### Measurement of soil properties

The soil was dried in a 105°C constant temperature drying oven (Thermo Fisher Scientific Corp., USA) for 12 h, and the difference in mass was considered a measure of the soil water content. Soil bulk density was determined according to the method described by Al-Shammary A (Al-Shammary et al., 2018). Soil pH was measured in a mixture with a soil-water ratio of 1/2.5 (wt/vol) using a pH meter (Thermo Fisher Scientific Corp., USA). The available states of copper, manganese, zinc, iron, and boron were extracted using DPTA and determined using atomic absorption spectroscopy (PerkinElmer, USA). Soil porosity was determined using the method described by David et al. (Moret-FernÁNdez and Lopez, 2019). Exchangeable calcium, magnesium, quick-acting potassium, and cations were replaced with ammonium acetate solution and then determined by atomic absorption spectroscopy (PerkinElmer, USA) and ion chromatography (Shimadzu, Japan). The available phosphorus content was extracted using sodium bicarbonate, colored with molybdenum antimony, and measured and calculated using a spectrophotometer (Shimadzu, Japan). The hydrolyzable nitrogen content was determined by steam distillation using 10 M NaOH after Kjeldahl digested of the acid hydrolysate (Lin et al., 2023). Soil organic matter content was measured using a CHNS/O elemental analyzer (Thermo Fisher Scientific Corp., USA).

### DNA sample extraction and Illumina sequencing

All DNA samples were extracted using the MP FastDNA spin kit according to the manufacturer’s instructions. The purity and concentration of total DNA were measured using a NanoDrop 2000 spectrophotometer (Thermo Fisher Scientific Corp., USA). PCR was performed using primer 341F (5’-CCTAYGGGRBGCASCAG-3’)/806R (5’-GGACTACNNGGGTATCTAAT-3’) to target the V3-V4 region of the 16S rRNA gene. The PCR reaction was performed with 25 μL 2x Premix Taq (TaKaRa Premix Taq® Version 2.0), 3 μL dNTP, 2 μL primers (10 μM), and 10 ng template DNA in 50 μL reaction systems. The thermal cycle of the 16S rRNA gene consists of an initial denaturation at 94°C for 5 min, followed by 30 cycles at 94°C for 30 s, 55°C for 30 s, 72°C for 30 s, and an extension at 72°C for 10 min. The fragment lengths and concentrations of the PCR products were measured through 1% agarose gel electrophoresis. Library construction was performed according to the standard procedure of NEBNext® Ultra™ II DNA Library Prep Kit for Illumina® (New England Biolabs, USA). The constructed amplicon library was sequenced using a PE250 on an Illumina Nova 6000 platform at Novogene Co., Ltd. (Beijing, China).

Low-quality data and primers were removed using FASTP (version 0.14.1) and Cutadapt software to obtain clean reads. Through the use of DADA2, the raw sequences were denoised and merged into a single sequence based on the overlapping region. Subsequent analyses were performed using a standard pipeline of Quantitative Insights into Microbial Ecology (QIIME, version 2). The representative sequences were annotated against the SILVA database (release 138). The ASVs annotated as chloroplasts or mitochondria (16S amplicons) that could not be annotated at the boundary level were removed for further analysis. Alpha diversity indices, including Chao 1, Shannon index, ACE, and good’s coverage were calculated. Differences in microbial community composition between the diseased and healthy samples were calculated using similarity analysis (ANOSIM). Non-metric multidimensional scaling (NMDS) analysis was performed using the Bray-Curtis distance algorithm. Significant differences between groups of species were analyzed using the Stamp software based on the relative abundance of ASVs. To determine the relationship between the environmental factors and microbial communities, redundancy analysis (RDA) was performed using Canoco (v5). Spearman’s rank correlation analysis was performed to determine the correlation between the relative abundance of dominant species and environmental factors. All the above analyses were performed using R software (v.3.5.3).

For the network analysis, rare ASVs (<0.01% of the total sequences) and specific genera (present in <1/3 of the total samples) were removed to reduce noise. Spearman’s correlation coefficients were calculated using a Molecular Ecological Network Analysis Pipeline (http://ieg2.ou.edu/MENA). Networks were constructed using random matrix theory-based methods. Network visualization and topological parameter analyses were performed using the Gephi software (v0.9.2).

### Metabolite extraction and sequencing

To identify metabolites with the potential to drive microbiome assembly and improve pathogen resistance in healthy plants, metabolites in the rhizosphere of healthy and diseased soils were extracted and analyzed according to the methods described by Wen et al. with some modifications(Wen et al., 2020). The soil samples were extracted with methanol solutions (methanol: chloroform =3:1, v/v) containing 20 μL L-2-chlorophenylalanine. The extract was dried in a vacuum concentrator, and 40 μL methoxamine salt reagent (methoxamine hydrochloride, dissolved in pyridine 20 mg/mL) was added to the dried metabolites, followed by incubation at 80°C for 30 min. Then, 60 BSTFA (containing 1% TMCS, v/v) reagent was added to each sample and the mixture was incubated at 70°C for 1.5 h. After incubation, 5 μL FAMEs (soluble in chloroform) were added to the mixed samples before being analyzed in a gas chromatograph device (Agilent 7890) combined with a Pegasus HT time-of-flight mass spectrometer. Mass spectral data were obtained in full scan mode, and the *m*/*z* range was 50-500.

### Metabolic data processing and analysis

Mass spectral data were analyzed using ChromaTOF software (V 4.3, LECO) for peak extraction, baseline correction, deconvolution, peak integration, and peak alignment. The LECO-Fiehn Rtx5 database was used for the qualitative analysis of substances. The peaks with a detection rate below 50% or RSD > 30% were excluded from further analysis. The collated data were logarithmically transformed and centrally formatted using SIMCA software (V15.0.2, Sartorius Stedim Data Analytics AB, Umea, Sweden), followed by automated modeling analysis and logarithmic transformation plus UV formatting. Differential metabolites were screened using a Student’s *t*-test with a *P*-value of less than 0.05. To characterise differences in metabolic composition, principal coordinate analysis (PCA) plots were generated from Bray-Curtis similarity matrices. A correlation analysis of the differential metabolites was performed using correlation calculations of the quantitative values of the differential metabolites for each set of comparisons. All pathways associated with the identified differential metabolites in the respective species were compiled using the Kyoto Encyclopedia of Genes and Genomes (KEGG) database. In the comprehensive analysis of metabolites, enrichment metabolites were included as factors to further refine the key pathways. Spearman’s rank correlation was performed using R (v. 3.5.3) to characterize the relationship between key metabolites and dominant genera.

## Results

### Soil properties

**Figure 1:**
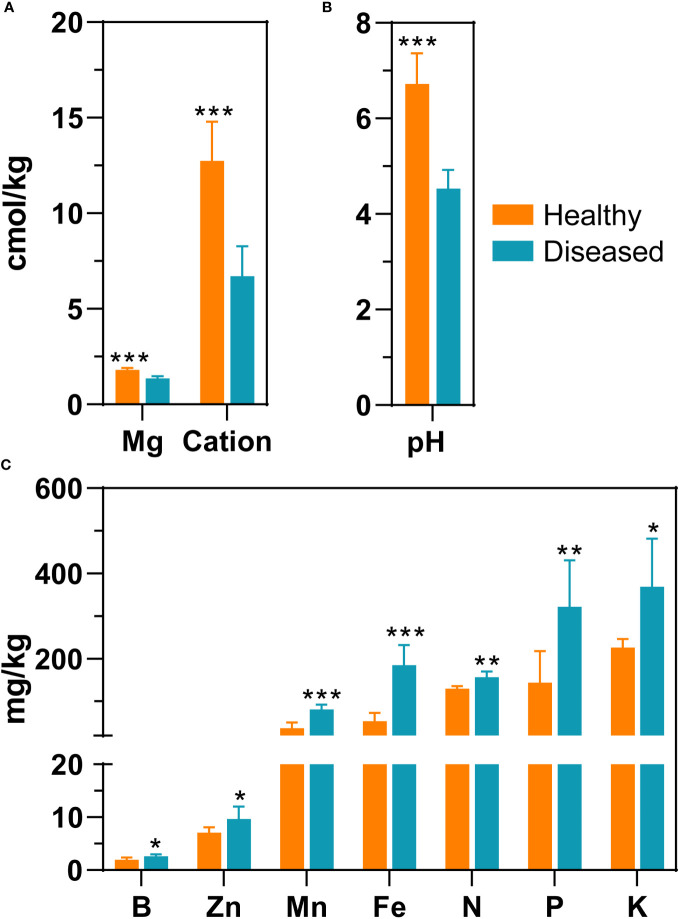
Differences in environmental parameters of rhizosphere soil samples from healthy plants and diseased plants. **(A)** Mg (exchangeable magnesium), cation (cation exchange capacity); **(B)** pH; **(C)** Zn (available zinc), B (available boron), Mn (available manganese), Fe (available iron), N (hydrolyzed nitrogen), P (available phosphorus), K (quick-acting potassium). The significance difference of soil properties between healthy plant and diseased plants was statistically analysed with a t-test (*, *P*<0.05; **, *P*<0.001; ***, *P*<0.0001).

In total, 12 rhizosphere soil samples were collected to determine soil properties, including six samples from diseased plants and six samples from healthy plants. Exchangeable magnesium, pH, and cation exchange capacity were significantly higher in healthy soil samples than in diseased soil samples (*P* < 0.05, *t*-test). Specifically, compared to diseased samples, the pH value of healthy samples was 2.2 units greater (6.73 ± 0.64 vs 4.53 ± 0.39) and the exchange magnesium content was 0.43 units greater (**Figures 1A, B** and **Supplementary Table 1**). The cation exchange capacity of the healthy group was two-fold greater compared to that of the diseased groups. In contrast, in the soil samples of diseased plants, seven environmental factors, including available manganese, available zinc, available iron, available boron, quick-acting potassium, hydrolyzable nitrogen, and available phosphorus, were significantly higher than the corresponding measures in the healthy group (*P* < 0.05, *t*-test). For example, the level of available iron was three times greater in the soil of diseased plants compared to the level of iron in the soil of healthy plants; similarly, the amount of available manganese and phosphorus was more than two times greater in the soil of diseased plants (**Figure 1C** and **Supplementary Table 1**).

### Diversity and composition of bacterial communities

**Figure 2:**
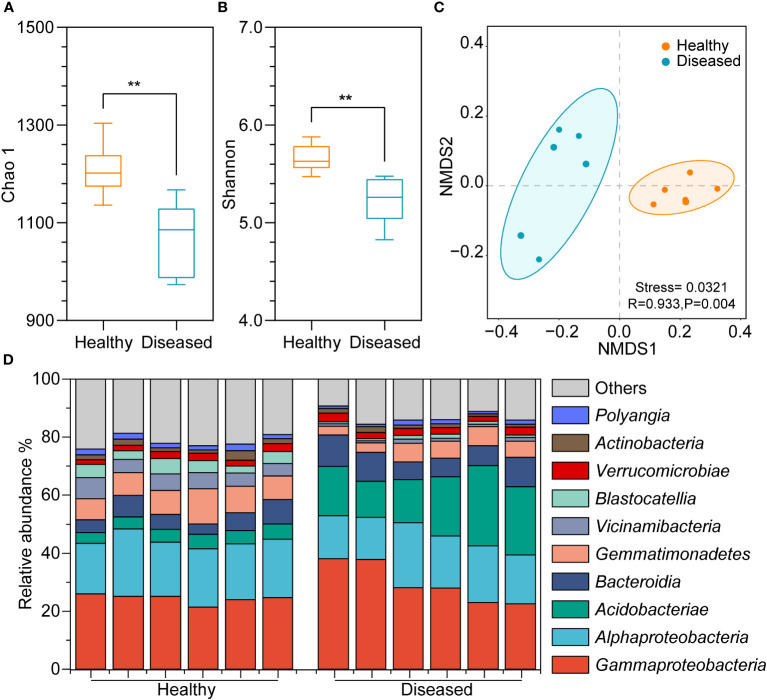
Diversity differences and community compositions of bacterial communities among different samples. **(A)** Chao 1 index; **(B)** Shannon index; **(C)** NMDS plot showing clustering relationship of bacterial communities between diseased and heathy groups, samples of different groups are color-coded; **(D)** Community composition of the top ten bacterial communities in different samples at the class level. (**, *P* <0.01; t-test).

Bacterial diversity was assessed via amplicon sequencing of the V3-V5 hypervariable regions of the 16S rRNA gene. A total of 5,143,167 sequences were obtained using 16S rRNA gene amplicon sequencing. To reduce the influence of sequencing depths on analysis results, the sequence numbers were rarefied according to the minimum sequences in the sample and 47,357 sequences remained in each sample. The total sequences clustering into 2,382 ASVs, ranging from 964 to 1294 in all samples (**Supplementary Table 1**). The good’s coverage of each sample was above 99.8%, indicating good representativeness of the sequencing data (**Supplementary Table 2**). The Chao1 results showed that the richness of bacterial species in the healthy group was significantly higher than in the diseased group (*P* < 0.05, *t*-test; **Figure 2A**). Additionally, the Shannon index was higher in the healthy group, suggesting a significantly greater bacterial diversity in the healthy group (*P* < 0.05, *t*-test; **Figure 2B**). The Pielou index revealed that, compared to the soil bacteria in the diseased tobacco rhizosphere (**Supplementary Table 2**), the number of bacteria in the rhizosphere of healthy plants was distributed more evenly within the community (*P* < 0.05). NMDS analysis revealed that bacterial communities were clustered based on the health conditions of plants (**Figure 2C**), and a significant difference in bacterial community structure between the rhizosphere soil of healthy and diseased plants was confirmed through ANOSIM analysis (*P* < 0.05).

A significant variance in the bacterial community composition was observed between the soil samples from healthy and diseased plants. In the healthy group, *Gammaproteobacteria*, *Alphaproteobacteria*, *Bacteroidia*, *Gemmatimonadetes*, and *Vicinamibacteria* were the dominant groups, comprising 21%-26%, 17%-23%, 4%-8%, 7%-12%, and 4%-7% of the bacterial community, respectively, whereas *Gammaproteobacteria* (23%-38%), *Alphaproteobacteria* (15%-22%), *Acidobacteriae* (12%-28%), and *Bacteroidia* (6%-11%) dominated in soil samples from diseased plants (**Figure 2D**). Additionally, *Alphaproteobacteria*, *Gemmatimonadetes*, *Vicinamibacteria*, and *Blastocatellia* were more abundant in the rhizosphere soil of healthy plants than in the rhizosphere of diseased plants, whereas *Gammaproteobacteria*, *Acidobacteriae*, and *Bacteroidia* were less abundant.

**Figure 3:**
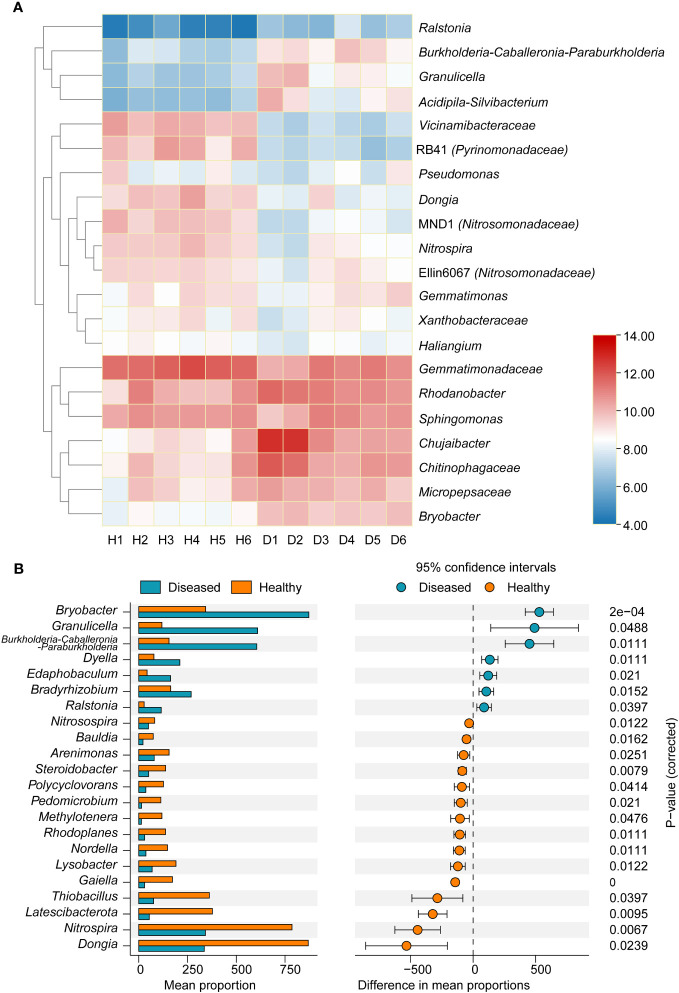
**(A)** Heatmap showing the relative abundance of dominant bacterial taxon (top 20) and *Ralstonia* genus at the genus level between two groups of samples. **(B)** Stamp analysis of the different of dominant bacterial genera between two groups. H, healthy plants; D, diseased plants.

At the genus level, marked differences were found between the diseased and healthy groups (**Figure 3A**). The relative abundance of the *Ralstonia* genus, which contains the pathogenic species of bacterial wilt disease, was significantly higher in the diseased group than in the healthy group (*P* < 0.05, *t*-test), which was consistent with the field phenotype. Compared with samples from healthy plants, there was a significant increase in the relative abundance of bacterial genera in the diseased group, including *Burkholderia*-*Caballeronia*-*Paraburkholderia*, *Granulicella*, *Acidipila*-*Silvibacterium*, and *Chitinophagaceae*. Conversely, the genera RB41 (*Pyrinomonadaceae*), *Dongia*, MND1 (*Nitrosomonadaceae*), and *Nitrospia* were significantly enriched in healthy samples. To explore the marker bacterial genera in different groups, we conducted a Stamp analysis of the top 0.1% of the dominant genera. The results showed that 15 bacterial genera, including *Dongia*, *Nitrospia*, *Latescibacterota*, *Thiobacillus*, etc., were significantly more abundant in the healthy group than in the diseased group (*P* < 0.05, **Figure 3B**), whereas *Bryobater*, *Granulicella*, *Burkholderia*-*Caballeronia*-*Paraburkholderia*, *Dyella*, *Edaphobaculum*, *Bradyrhizobium*, and *Ralastonia* were significantly enriched in the disease groups.

### Changes in microbial networks under pathogen invasions

**Figure 4:**
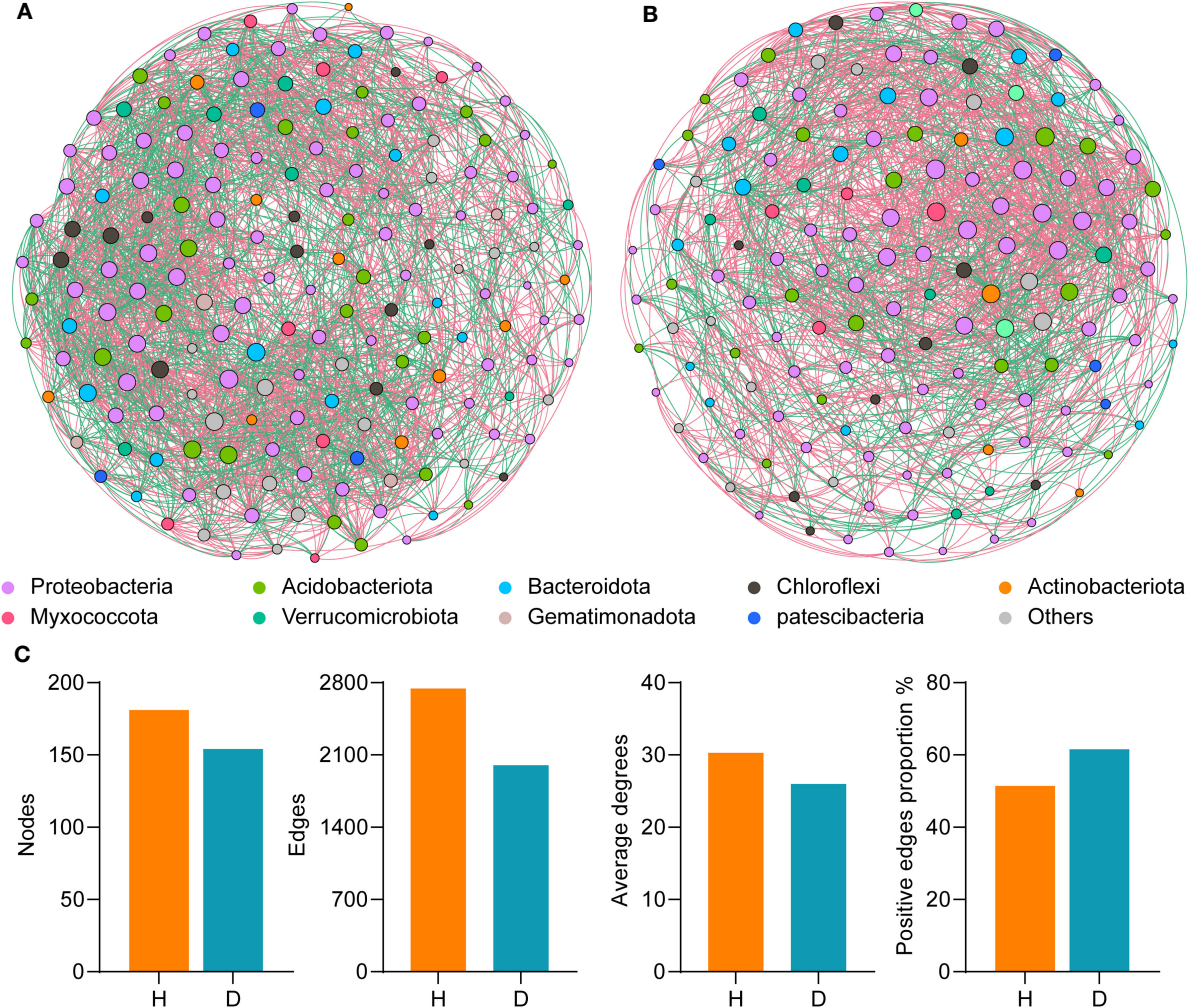
Co-occurrence network analysis of healthy and diseased tobacco plants. **(A)** The symbiotic network of microbial communities in healthy rhizosphere soil samples **(B)** The symbiotic network of microbial communities in diseased rhizosphere soil samples. The node is coloured at the class level, and the size of nodes represents the node degree of genera; **(C)**. Comparison of network topological parameters between healthy and diseased samples. H, healthy plants; D, diseased plants.

To characterize the microbial networks in the rhizospheres of healthy and diseased plants, rare species at the genus level were removed from analysis (0.1‰ of the total sequences). Spearman’s correlation analysis was conducted to assess the co-occurrence patterns among bacterial communities in the different samples. Significant differences in the structural and topological characteristics of the networks were observed between the diseased and healthy groups (**Figure 4** and **Supplementary Table 3**). In the rhizosphere soil of healthy plants, the bacterial community network consisted of 181 nodes, 2,742 connections, and an average degree of 30.298, which was greater than the corresponding results in the samples from diseased plants, which contained 154 nodes, 2,000 connections, and an average degree of 25.97. The number of positive connections was higher in the disease group than in the healthy group. Furthermore, the centralization of stress centrality (CS) in the healthy group was higher (0.458) than in the diseased rhizosphere soil (0.262). These results indicated that the bacterial community forms a more highly interactive and complex network in the rhizosphere soil of healthy plants than in that of diseased plants.

Based on the criteria of node degree and betweenness centrality, genera with the top 20% node degree and betweenness centrality values and the bottom 20% were regarded as keystone taxa. In the rhizosphere microbiome of healthy plants, seven genera, *Sphingomonas*, *Bryobacter*, *Micropepsis*, *Phenonobacterium*, *Acidipila, Silvibacterium*, and *Pseudomonas*, were the keystone taxa (**Supplementary Table 4**). These bacterial groups may be involved in maintaining community stability and in the prevention and control of bacterial wilt. In the rhizosphere microbiome of diseased plants, *Acidipila, Silvibacterium*, *Sphingomonas*, *Dechromonas*, *Chujaibacter*, *Bacillus*, and *Hyphomicrobium* were the keystone taxa.

### Key environmental factors affecting bacterial communities

Redundancy analysis (RDA) was conducted to identify environmental factors affecting community structure. RDA1 explained 62.00% of the total variation, whereas RDA2 explained 19.58% (**Supplementary Figure S1**). Exchangeable magnesium, cation exchange capacity, and pH were found to be the key environmental factors affecting the bacterial community in diseased rhizosphere soils, whereas available phosphorus, available manganese, hydrolyzable nitrogen, available iron, and quick-acting potassium were the main factors affecting the bacterial community structure in the soils of healthy plants.

Spearman’s correlation analysis was conducted between the dominant bacterial genera and environmental factors (**Supplementary Figure S2**), and it was found that *Ralstonia* genus showed a significant negative correlation with pH, exchangeable magnesium, and cation exchange flux and a positive correlation with available iron. Furthermore, bacterial genera significantly enriched in the diseased group, namely *Burkholderia*-*Caballeronia*-*Paraburkholderia*, *Granulicella*, *Acidipila*-*Silvibacterium*, and *Chitinophagaceae*, also exhibited negative correlations with pH, exchangeable magnesium, and cation exchange flux, and positive correlations with available phosphorus, available manganese, hydrolyzable nitrogen, available iron, and quick-acting potassium levels. Conversely, bacterial genera that were significantly enriched in the healthy group, namely RB41, *Dongia*, MND1, and *Nitrospia*, were positively correlated with pH, exchangeable magnesium, and cation exchange flux, and negatively correlated with available phosphorus, available manganese, hydrolyzable nitrogen, available iron, and quick-acting potassium levels.

### Changes in metabolites among different samples

**Figure 5:**
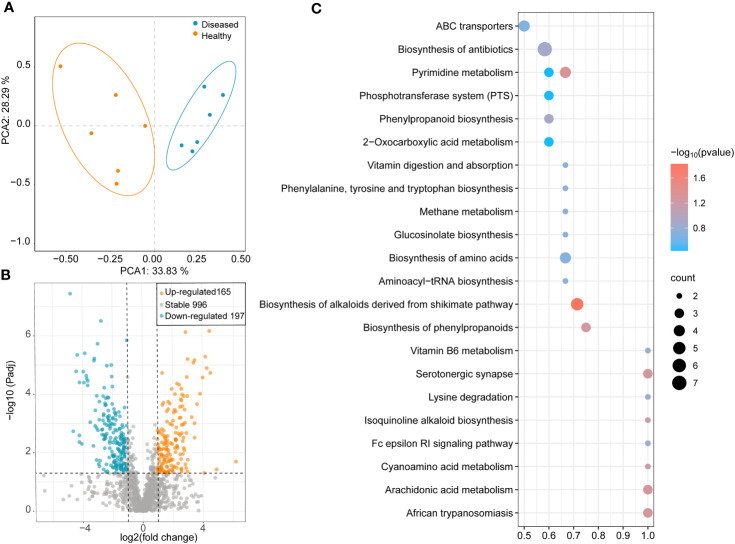
**(A)** PCA analysis of different samples based on bray-cutis-distance. **(B)** Volcano Plot shows the differential metabolites between healthy and diseased plant rhizosphere soil samples. **(C)** Heatmap shows functional differences in KEGG enrichment.

The results of the PCA showed that all samples were clustered separately based on the healthy conditions of the plants, with two principal components explaining 62.12% of the overall variance (33.83% and 28.29% for PCA1 and PCA2, respectively; **Figure 5A**). In the healthy group, 352 metabolites showed significant changes: 165 metabolites were significantly upregulated, and 197 metabolites were significantly downregulated (**Figure 5B**). To identify the key metabolites participating in the defense against pathogens, a KEGG pathway enrichment analysis was performed. The results showed that, compared to the rhizosphere metabolites of diseased plants, several pathways were significantly enriched in the rhizosphere samples of healthy plants, including ABC transporters, biosynthesis of antibiotics, phenylpropanoid biosynthesis, vitamin B6 metabolism, biosynthesis of alkaloids derived from shikimate pathway, pyrimidine metabolism, biosynthesis of phenylpropanoids, and isoquinoline alkaloid biosynthesis (**Figure 5C**). Seven genes were significantly enriched in pathways related to antibiotic biosynthesis (**Figure 5C**).

**Figure 6:**
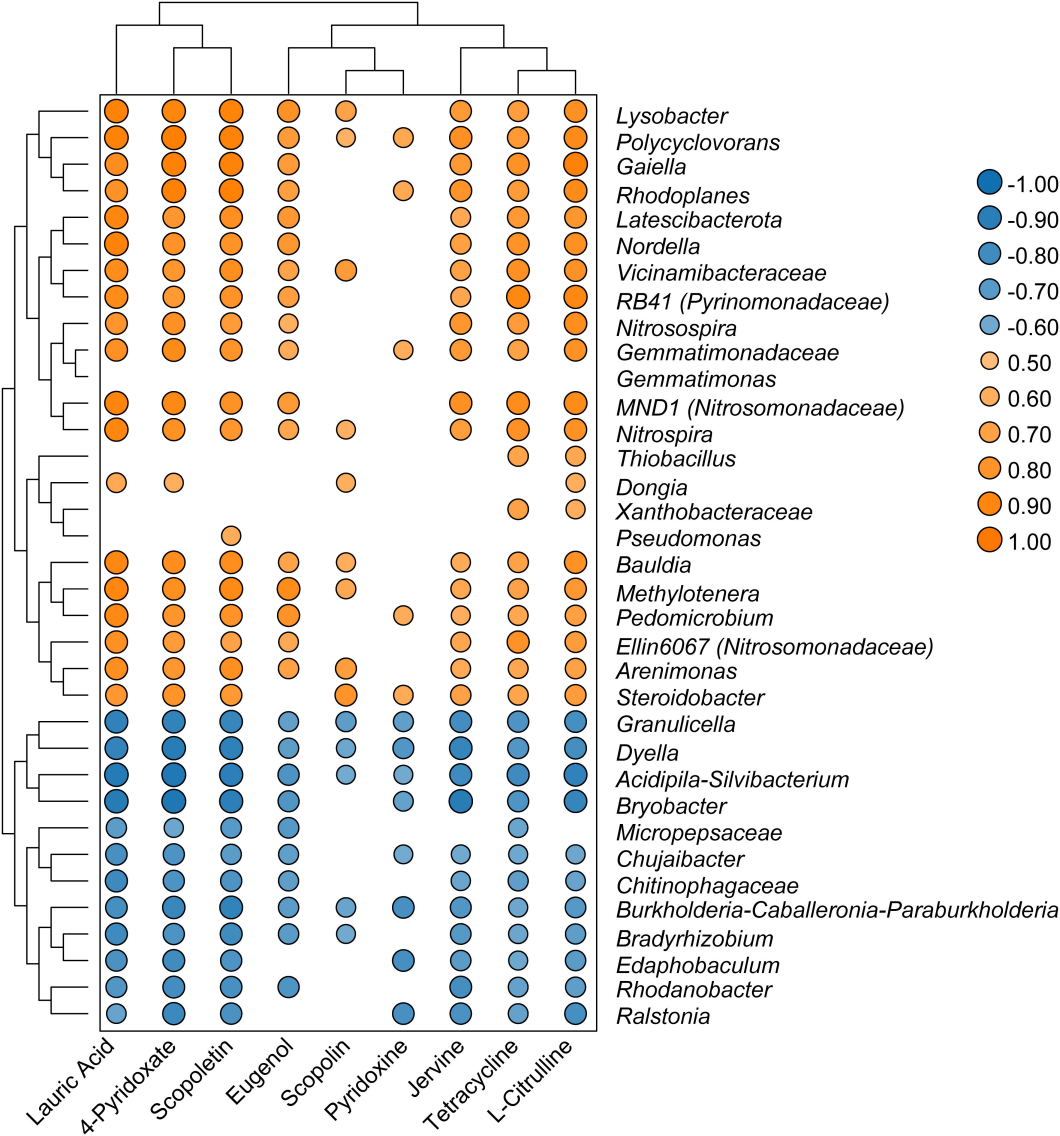
Heatmap showed that correlations between selected genera and significant changed metabolites.

Spearman’s analysis was performed using the top 35 genera with significant changes in relative abundance and nine differential metabolites associated with antimicrobial pathways. These nine metabolites were lauric acid, tetracycline, scopolin, eugenol, scopoletin, jervine, l-citrulline, 4-pyridoxate, and pyridoxine. The results showed that the *Ralstonia* genus was negatively correlated with seven metabolites, whereas *Polyclovorans* was positively correlated with all nine metabolites (**Figure 6**). Scopoletin was positively correlated with the bacterial genera RB41, *Gaiella*, Ellin6067, MND1, *Pseudomonas*, *Nitrosospira*, *Polycyclovorans*, and *Lysobacter*, indicating that scopoletin (known to have antibacterial activity) may be generated by these bacterial taxa. Moreover, tetracycline, which is used as a broad-spectrum antibiotic, was positively correlated with bacteria belonging to the genera RB41, *Gaiella*, *Ellin6067*, MND1, *Nitrosospira*, *Polycyclovorans*, and *Lysobacter*. The metabolites 4-pyridoxate and pyridoxine, which are related to vitamin B6 metabolism, were also positively correlated with these bacterial genera.

## Discussion

The microbial community in plant rhizosphere soil is crucial for resisting pathogen invasion and maintaining plant health (Kashyap et al., 2022). At present, most research focuses on exploring the functions and effects of individual biocontrol strains; however, the mechanisms by which the microbial community exerts its effects in the field are still unclear. Here we analyzed the changes in soil properties, bacterial community responses, and metabolomics in the rhizosphere of tobacco plants infected with bacterial wilt. We found significant differences in the soil environmental parameters between the diseased and healthy samples. The occurrence of bacterial wilt significantly reduced the diversity of soil microbial communities and had a significant effect on the network structures of the microbial communities. Metabolic pathways related to the synthesis of various antibacterial compounds that may participate in pathogen resistance were significantly enriched in the healthy group, and potential producers of these compounds were identified. Our findings provide a strong theoretical basis for the effective utilization of soil microbial communities for the biocontrol of crop diseases in the field.

### Rhizosphere microbiomes were influenced by bacterial wilt

In the study, the relative abundance of *Ralstonia* genus in diseased plant samples significantly increased. The relative abundance of the *Ralstonia* genus from amplicon sequencing was been reported to be correlated positively with the density of pathogenic *R. solanacearum* determined (Zheng et al., 2021). The observed increase in *Ralstonia* genus abundance in the diseased group might confirm the occurrence of bacterial wilt. The diversity and community composition of rhizosphere microbiomes were influenced by the bacterial wilt. Soil microbial communities are closely associated with plant health, and high microbial diversity plays an important role in maintaining plant health (Berg et al., 2017). The more abundant bacterial genera and microbial diversity (Shannon index) were found in soil samples of healthy plants than in soil samples of diseased plants, which is consistent with previous findings (Niu et al., 2016). When tobacco plants are infected with bacterial wilt pathogens, they may cause the rapid growth of dominant species that outcompete other bacteria for survival space, thereby restricting the growth of other microbial groups. In addition, the evenness of the community (Pielou index) was higher in healthy samples than in diseased samples, indicating better diversity structure and a more stable community in healthy groups. Moreover, the NMDS and ANOSIM analysis confirmed the variation of bacterial assemblages by revealing a clear community separation according to plant health. This was consistent with previous reports which found bacterial and eukaryote communities were changed with invasion of pathogens(Gao et al., 2021).

In soil microbial system, the functionality of the microbial community is not merely the sum of the functions of individual microbial taxa. There are also frequent interactions between various microorganisms (Van der Heijden and Hartmann, 2016). Agler et al. (Agler et al., 2016) found that the plant host genotype can influence key microbial species, modulate interactions among microorganisms, and alter host adaptability, thereby affecting the entire microbial community. In this study, the number of nodes, connections, and average path lengths in the network were significantly higher in soil bacterial networks associated with healthy plants compared to those in soils from diseased plants, which is consistent with previous research results (Wei et al., 2018). The number of positive connections was higher in the disease group, indicating that there is more synergistic effect between microbial populations. In the diseased plant, there might be more microbiome with growth-promotive siderophores, causing a decrease in competition (Gu et al., 2020). Additionally, the microbial community in the rhizosphere of healthy plants exhibits a high degree of centralization of stress (CS), indicating more microbial hubs in the network may play a central role in responding to environmental stress and defending against the invasion of pathogens (Runge et al., 2023). Collectively, close interactions between different species may enhance effective defense against invading pathogens, and a more intricate microbial network might be pivotal for disease resistance in healthy root systems (Carrión et al., 2019).

Keystone taxa occupying central positions interact more closely with various community groups and play a crucial role in maintaining soil microenvironmental systems and regulating plant growth (Banerjee et al., 2018). As keystone taxon in the healthy tobacco rhizosphere soil, bacterial taxa such as *Sphingomonas*, *Silvibacterium*, and *Pseudomonas* are of great significance for the stability of bacterial co-occurrence network and the healthy growth of plants. For example, *Sphingomonas* significantly promotes plant growth and contributes to the degradation of persistent metabolites in the environment (Asaf et al., 2020). Previous studies have shown that *Pseudomonas* has a significant antagonistic ability against pathogenic bacteria and a significant inhibitory effect on bacterial wilt (Nadhira et al., 2021). However, *Acidipila*, *Silvibacterium*, *Sphingomonas*, and *Bacillus* were keystone taxons in the rhizosphere soil of diseased tobacco. *Acidipila* and *Silvibacterium* are both *Acidobacteria*, which have growth advantages under low pH conditions in diseased soil (Zhang et al., 2022). In addition, when plants are infected with pathogens, the structure and composition of the rhizosphere microbial community undergo significant changes (Diaz-Cruz and Cassone, 2022). Plants can recruit microbial communities during pathogen attacks and create a long-lasting protective soil microbial community (Goossens et al., 2023). *Sphingomonas* and *Bacillus*, as keystone taxon in the diseased rhizosphere soil, may play a role in responding to plant resistance to pathogen infection. The absence of these keystone taxa will make plants more vulnerable and difficult to adapt to external environmental changes and stress (Yin et al., 2022). Moreover, significant enrichment of bacteria such as MND1, *Gaiella*, *Rhodoplanes*, *Nitrospia*, *Latescibacterota*, and *Thiobacillus* was observed in healthy soil samples. These bacteria may be involved in functions related to plant-root symbiosis, nutrient cycling, and organic matter decomposition, thereby contributing positively to plant health (Ahmed et al., 2022). For example, MND1 is an ammonia-oxidizing bacterium that affects nutrient absorption in plants (Wang et al., 2022c) and inhibits pathogens (Yang et al., 2023b). *Dongia* is considered to be involved in the soil nitrogen cycle, which may synergistically improve soil available nutrients and root uptake, promoting plant growth (Wang et al., 2022a; Sanka Loganathachetti and Mundra, 2023). *Nitrospira* was reported to be involved in the degradation and cycling of soil organic matter and increasing soil pH, thereby improving plant stress resistance of soil acidification and nutrient absorption (Hu et al., 2021). Although the specific functions of these genera and their interactions with plants may vary depending on the characteristics of the strains, plant species, and environmental conditions, these findings indicated that members of the rhizosphere microbiota have a significant impact on plant metabolism and resistance to pathogens (Le et al., 2022).

Soil physicochemical properties play a crucial role in the formation of soil microbial community structure (Liang et al., 2018). We found that pH, exchangeable magnesium, and cation exchange capacity are important environmental factors affecting the soil microbial community in the diseased plant rhizosphere. This is consistent with previous reports that a significant decrease in pH in the rhizosphere soil of diseased plants leads to weakened tobacco resistance, affecting microbial community structure (Qi et al., 2019). Higher soil pH directly influences plant disease infection by affecting the growth and reproduction of plant pathogens (Shen et al., 2018). In addition, lower soil pH can lead to a decrease in soil exchangeable magnesium content, significantly affecting the photosynthesis, enzyme activation, and metabolic reactions of tobacco (Farhat et al., 2016). The decrease in cation exchange capacity directly leads to a decrease in the ability of the soil to retain essential nutrients affecting the growth of plants and reducing their resistance to disease (Arthur, 2017). Therefore, pH and cation exchange capacity could be regarded as a potential environmental indicator for the onset of bacterial wilt disease. Furthermore, the amounts of available phosphorus, manganese, hydrolyzable nitrogen, iron, and quick-acting potassium significantly increase in diseased soil, which may indirectly affect plant health (Sun et al., 2022). For example, phosphorus availability has been found to increase the abundance of pathological biota, thereby increasing the infection by *Ralstonia* in the rhizosphere of plants (Li et al., 2021). A large amount of nitrogen and phosphorus in the soil may lead to a decrease in soil microbial biomass and changes in the structure of rhizosphere microbial communities (Wang et al., 2022e).

### Specific rhizosphere metabolites in different plants

Metabolites in the soil play a crucial role in regulating plant-microbe interactions (Bi et al., 2021), and significant metabolite enrichment primarily occurs in the rhizosphere (Wong-Bajracharya et al., 2020). Similar to the results of Zhao et al., the results we report here indicated significant differences in the metabolites between the rhizospheres of diseased and healthy plants (Zhao et al., 2023). Compared to the metabolic profiles of rhizosphere soils from diseased plants, soils from healthy plants exhibited significant enrichment in metabolic pathways related to the synthesis of antimicrobial substances. These pathways include the biosynthesis of alkaloids derived from the shikimate pathway, phenylpropanoids, isoquinoline alkaloids, and antibiotics. This indicated that the synthesis of various antibiotics and alkaloids in the rhizosphere soil of healthy plants may effectively inhibit the growth of pathogenic microorganisms (Molina-Santiago et al., 2015). Furthermore, various metabolic pathways with important physiological functions in plants were significantly enriched in the rhizosphere of healthy plants (**Figure 5C**). For example, pyrimidine metabolism is closely associated with the synthesis of DNA, RNA, lipids, and carbohydrates (Garavito et al., 2015). ABC transporters participate in the active transport of intracellular substances, regulate the absorption, excretion, and distribution of substances, and participate in the biosynthesis of chlorophyll (Martinoia et al., 2014). Compounds produced in the phenylpropanoid and phenylalanine biosynthetic pathways have diverse physiological functions in plants, including antioxidation, defense, and signal transduction activities. The phosphotransferase system (PTS) in bacteria plays a crucial role in regulating sugar absorption and utilization, carbohydrate transport, and metabolism, thereby significantly impacting bacterial growth and metabolism (Wang et al., 2022d). These metabolic pathways are interconnected and collectively maintain the metabolic balance and physiological processes within organisms. Notably, while these metabolic pathways are primarily present in plants, similar biosynthetic pathways or related metabolic pathways may also exist in some microorganisms that synthesize similar compounds (Perry et al., 2022). Rhizosphere microorganisms and plants have been shown to interact and connect by producing corresponding secretions and hormones (De Vries et al., 2020). Although plants regulate the rhizosphere microbial ecosystem through corresponding metabolic and signaling pathways, rhizosphere microorganisms also influence plant growth through the production of metabolites.

The microbial community is an important factor driving the distribution of soil metabolites, and bacteria dominate the symbiotic network between microbial members and metabolites (Li et al., 2020). The *Ralstonia* genus exhibited negative correlations with seven active metabolites: lauric acid, jervine, scopoletin, tetracycline, 4-pyridoxate, l-citrulline, and pyridoxine. These antibiotic substances may directly participate in defense against pathogens. For example, tetracycline, lauric acid, and jervine can inhibit protein biosynthesis and block signal transduction in pathogens (Schäfer, 2017; Chen et al., 2020; Welch et al., 2020). Pyridoxine and 4-pyridoxate are directly related to vitamin B6 levels. Although vitamin B6 does not have a direct antibacterial effect, it can help plants cope with various environmental stresses such as drought, salt stress, and diseases. Pyridoxine acts as an antioxidant in plants and interferes with pathogen defense responses (Fudge et al., 2017). This indicate that secondary metabolites in the soil have many important ecological roles and can be used as bactericides to inhibit plant pathogens (Guo et al., 2022). In contrast, several genera that were significantly enriched in the healthy group samples, such as RB41 (*Pyrinomonadaceae*), *Gaiella*, Ellin6067 (*Nitrosomonadaceae)*, *Pseudomonas*, MND1 (*Nitrosomonadaceae*), *Polycyclovorans*, and *Lysobacter*, were significantly and positively correlated with these nine key metabolites, suggesting their potential involvement in the synthesis of antibiotics and alkaloids. Therefore, these enriched bacterial taxa may be involved in maintaining soil health in tobacco fields. Rhizosphere antibacterial compounds can selectively promote the growth of specific microorganisms by restricting certain microorganisms, thus providing a mutually beneficial balance in the interaction between plants and microorganisms (Dastogeer et al., 2020). Therefore, the use of microbiomes with antibiotic abilities may reduce chemical pesticide use and lead to more sustainable control of soil-borne pathogens. In the future, the effects of keystone bacteria on pathogen invasion should be evaluated more comprehensively using physiological and biochemical analyses, greenhouse, and field experiments of a single isolate.

## Conclusion

The occurrence of soil-borne plant diseases is closely related to the stability (and instability) of rhizosphere microbiota. In this study, we investigated the microbial community composition and metabolic profiles of the tobacco rhizosphere during the invasion of *R. solanacearum*, revealing the distinct microbial structures and potential defense strategies of plants. The high diversity and complex occurrence networks of tobacco rhizosphere microorganisms are important for plant rhizosphere health. In addition, environmental parameters, such as soil pH, cation exchange capacity, and exchangeable magnesium, are key factors influencing microbial community structure and pathogen growth. Lauric acid, tetracycline, eugenol, scopolin, jervine, L-citrulline, scopoletin, 4-pyridoxate, and pyridoxine are key metabolites involved in maintaining plant health. Potential bacterial groups involved in the synthesis of key metabolites were identified, namely *Pseudomonas*, *Gaiella*, *Nitrobacteria*, *Polycyclovorans*, and *Lysobacteria*. The symbiotic relationship between plants and microorganisms plays an important role in the formation and function of plant rhizosphere ecosystems, jointly resisting the invasion of bacterial wilt and promoting the healthy growth of plants. These findings may help understand the disease resistance mechanism of rhizosphere bacteria in the initial stage of disease infection, and samples of different disease incidence levels need to be investigated in the future to obtain the dynamic response mechanisms of rhizosphere microorganisms to whole infection stages.

## Data availability statement

The datasets presented in this study can be found in online repositories. The names of the repository/repositories and accession number(s) can be found below: https://www.ncbi.nlm.nih.gov/,PRJNA1033381.

## Author contributions

CW: Data curation, Investigation, Writing – original draft. JL: Data curation, Funding acquisition, Investigation, Writing – review & editing. RW: Conceptualization, Investigation, Writing – review & editing. LC: Data curation, Writing – review & editing. WW: Investigation, Writing – review & editing. JT: Investigation, Writing – review & editing. HS: Investigation, Writing – review & editing. XS: Data curation, Writing – review & editing. ZC: Data curation, Writing – review & editing. QX: Data curation, Writing – review & editing. DC: Funding acquisition, Writing – original draft. XW: Conceptualization, Funding acquisition, Writing – review & editing.
