# Supplementary material for: Response of bacterial community metabolites to bacterial wilt caused by Ralstonia solanacearum: a multi-omics analysis
Source: Front Plant Sci. 2024 Jan 22;14:1339478. doi: 10.3389/fpls.2023.1339478 (PMC10839043; doi:10.3389/fpls.2023.1339478)
Supplement: Supplementary file 1 [file DataSheet_1.docx]

**Response of bacterial community metabolites to bacterial wilt caused by *Ralstonia solanacearum*: A multi-omics analysis**


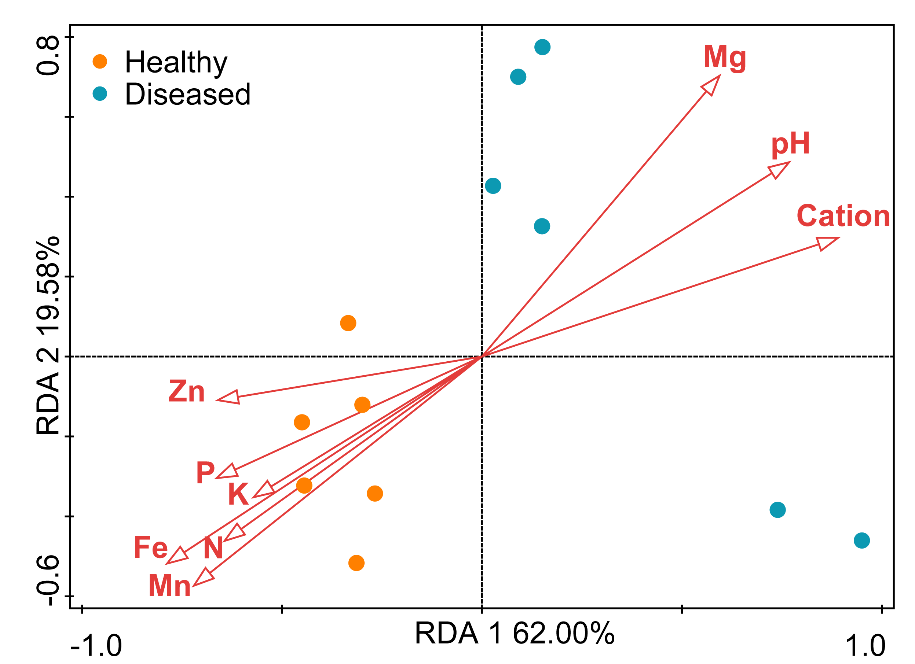


Figure S1 Redundancy analysis (RDA) between different samples. H, Healthy samples; D, diseased samples. Zn (available zinc), Mg (exchangeable magnesium), cation (cation exchange capacity), Mn (available manganese), Fe (available iron), N (hydrolyzable nitrogen), P (available phosphorus), K (available potassium).


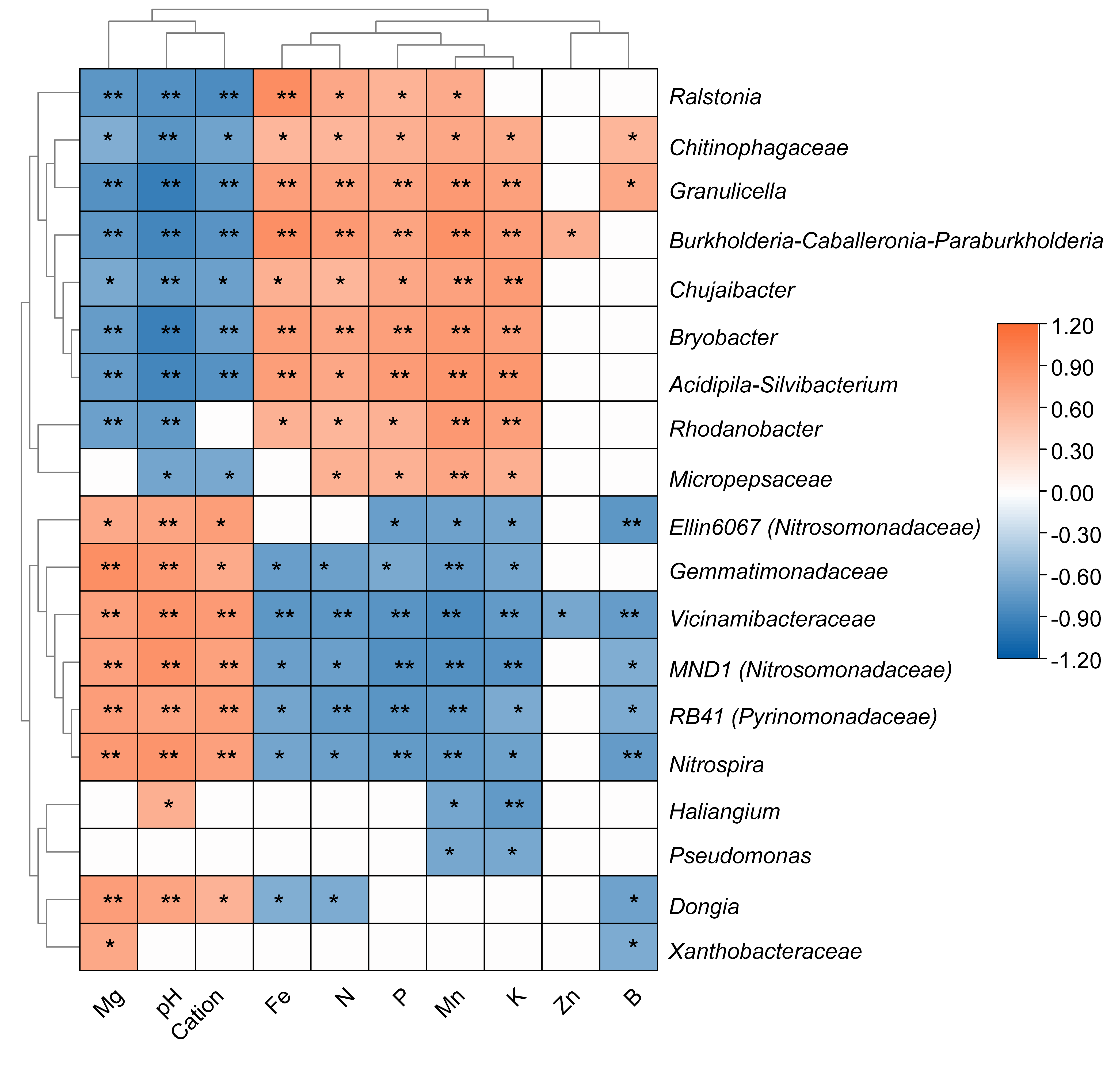


Figure S2 Spearman correlation analysis was conducted between dominant bacterial genera and environmental factors. Zn (available zinc), Mg (exchangeable magnesium), cation (cation exchange capacity), Mn (available manganese), Fe (available iron), N (hydrolyzable nitrogen), P (available phosphorus), K (available potassium). (_*_, *P*<0.05, _**_ *P*<0.001, _***_, *P*<0.0001)

Table S1 Environmental parameters of samples

| Number | Healthy | Diseased | *P-*value |
| --- | --- | --- | --- |
| Water content % | 15.39±1.4 | 15.57±0.84 | 0.788 |
| Unit weight g/cm3 | 1.17±0.06 | 1.15±0.08 | 0.6179 |
| Total porosity % | 55.4±1.71 | 55.92±2.37 | 0.6719 |
| Capillary water capacity % | 18.21±2 | 18.45±1.17 | 0.8055 |
| Capillary porosity % | 21.26±2.34 | 21.22±1.25 | 0.9724 |
| Ventilation porosity % | 34.14±3.2 | 34.7±3.17 | 0.7679 |
| pH | 6.73±0.64 | 4.53±0.39 | **0.0001** |
| Available copper mg/kg | 1.86±0.43 | 2.05±0.54 | 0.4283 |
| Available manganese mg/kg | 37.37±13.54 | 81.47±11.43 | **0.0001** |
| Available mg/kg | 7.1±1.03 | 9.67±2.35 | **0.0129** |
| Available iron mg/kg | 54.25±19.13 | 185.67±46.55 | **0.0001** |
| Available boron mg/kg | 1.94±0.45 | 2.48±0.48 | **0.034** |
| Exchangeable calcium cmol/kg | 5.62±2.53 | 3.72±0.18 | 0.0504 |
| Exchangeable magnesium cmol/kg | 1.8±0.11 | 1.37±0.11 | **0.0001** |
| Quick-acting potassium mg/kg | 226±20.38 | 368±113.13 | **0.0037** |
| Hydrolyzable nitrogen mg/kg | 130±6.23 | 156±13.9 | **0.0006** |
| Available phosphorus mg/kg | 144.3±73.73 | 321.97±108.9 | **0.0021** |
| Organic matter g/kg | 20.93±8.38 | 27.27±6.63 | 0.8046 |
| Cation exchange capacity cmol(+)/kg | 12.73±2.07 | 6.72±1.56 | **0.0001** |

Note: Significant differences are indicated in bold.

Table S2 Diversity indices of bacterial communities of healthy (H) and diseased (D) samples

|  | Richness | Shannon | Simpson | Pielou | invsimpson | Chao1 | ACE | goods_coverage | ASV number |
| --- | --- | --- | --- | --- | --- | --- | --- | --- | --- |
| D1 | 954 | 4.827272 | 0.969187 | 0.703616 | 32.45412 | 991.2273 | 996.3015 | 0.998078 | 966 |
| D2 | 1078 | 5.107594 | 0.974641 | 0.731447 | 39.43378 | 1112.964 | 1115.849 | 0.998121 | 1088 |
| D3 | 1142 | 5.435865 | 0.988061 | 0.772081 | 83.76231 | 1167.443 | 1172.572 | 0.998374 | 1150 |
| D4 | 1094 | 5.476772 | 0.989346 | 0.782665 | 93.86502 | 1116.619 | 1126.265 | 0.998395 | 1102 |
| D5 | 957 | 5.149125 | 0.984603 | 0.750185 | 64.9486 | 973.2614 | 975.9477 | 0.99886 | 964 |
| D6 | 1035 | 5.372956 | 0.987635 | 0.773961 | 80.87037 | 1058 | 1061.439 | 0.998543 | 1041 |
| H1 | 1186 | 5.755379 | 0.991475 | 0.813097 | 117.2987 | 1203.353 | 1207.07 | 0.998733 | 1193 |
| H2 | 1161 | 5.634745 | 0.989809 | 0.798458 | 98.1245 | 1186.235 | 1184.579 | 0.998606 | 1164 |
| H3 | 1191 | 5.623083 | 0.989481 | 0.793935 | 95.06679 | 1217.129 | 1222.696 | 0.99829 | 1203 |
| H4 | 1120 | 5.471562 | 0.986294 | 0.779304 | 72.95987 | 1136.162 | 1143.097 | 0.998691 | 1122 |
| H5 | 1290 | 5.879384 | 0.991724 | 0.820868 | 120.8343 | 1303.531 | 1306.75 | 0.998902 | 1294 |
| H6 | 1179 | 5.588207 | 0.989356 | 0.79014 | 93.95195 | 1200.45 | 1203.092 | 0.998606 | 1183 |

Table S3 Overall Topology Parameters of the Network Diagram

| Network Indexes | Diseased | Healthy |
| --- | --- | --- |
| Total nodes | 154 | 181 |
| Total links | 2000 | 2742 |
| R square of power-law | 0.029 | 0.082 |
| Average degree (avgK) | 25.974 | 30.298 |
| Average clustering coefficient (avgCC) | 0.498 | 0.507 |
| Average path distance (GD) | 2.19 | 2.187 |
| Geodesic efficiency (E) | 0.526 | 0.525 |
| Harmonic geodesic distance (HD) | 1.903 | 1.903 |
| Maximal degree | 51 | 57 |
| Nodes with max degree | asv085 | ASV009 |
| Centralization of degree (CD) | 0.166 | 0.15 |
| Maximal betweenness | 264.136 | 326.077 |
| Nodes with max betweenness | asv114 | ASV177 |
| Centralization of betweenness (CB) | 0.015 | 0.014 |
| Maximal stress centrality | 4979 | 10831 |
| Centralization of stress centrality (CS) | 0.262 | 0.458 |
| Maximal eigenvector centrality | 0.165 | 0.152 |
| Centralization of eigenvector centrality (CE) | 0.103 | 0.094 |
| Density (D) | 0.17 | 0.168 |
| Reciprocity | 1 | 1 |
| Transitivity (Trans) | 0.53 | 0.527 |

Table S4 Keystone taxa identified in the co-occurrence network of healthy (H) and diseased (D) group

| ID | Degree | Betweenness centrality | Taxonomy |
| --- | --- | --- | --- |
| D-1 | 40 | 63.90454954 | d__Bacteria;p__Acidobacteriota;c__Acidobacteriae;o__Acidobacteriales;f__Acidobacteriaceae;g__Acidipila-Silvibacterium |
| D-2 | 42 | 70.96017641 | d__Bacteria;p__Proteobacteria;c__Alphaproteobacteria;o__Sphingomonadales;f__Sphingomonadaceae;g__Sphingomonas |
| D-3 | 42 | 70.96017641 | d__Bacteria;p__Proteobacteria;c__Gammaproteobacteria;o__Burkholderiales;f__Rhodocyclaceae;g__Dechloromonas |
| D-4 | 38 | 71.57330503 | d__Bacteria;p__Proteobacteria;c__Gammaproteobacteria;o__Xanthomonadales;f__Rhodanobacteraceae;g__Chujaibacter |
| D-5 | 44 | 90.26428777 | d__Bacteria;p__Firmicutes;c__Bacilli;o__Bacillales;f__Bacillaceae;g__Bacillus |
| D-6 | 40 | 97.79367736 | d__Bacteria;p__Proteobacteria;c__Alphaproteobacteria;o__Rhizobiales;f__Hyphomicrobiaceae;g__Hyphomicrobium |
| D-7 | 38 | 103.9795004 | d__Bacteria;p__Proteobacteria;c__Gammaproteobacteria;o__Burkholderiales;f__Comamonadaceae;g__Ramlibacter |
| H-1 | 47 | 111.8617939 | d__Bacteria;p__Proteobacteria;c__Alphaproteobacteria;o__Sphingomonadales;f__Sphingomonadaceae;g__Sphingomonas |
| H-2 | 47 | 119.118131 | d__Bacteria;p__Acidobacteriota;c__Acidobacteriae;o__Bryobacterales;f__Bryobacteraceae;g__Bryobacter |
| H-3 | 47 | 119.118131 | d__Bacteria;p__Proteobacteria;c__Alphaproteobacteria;o__Micropepsales;f__Micropepsaceae;g__Micropepsis |
| H-4 | 47 | 119.118131 | d__Bacteria;p__Proteobacteria;c__Alphaproteobacteria;o__Caulobacterales;f__Caulobacteraceae;g__Phenylobacterium |
| H-5 | 44 | 123.8980422 | d__Bacteria;p__Acidobacteriota;c__Acidobacteriae;o__Acidobacteriales;f__Acidobacteriaceae;g__Acidipila-Silvibacterium |
| H-6 | 51 | 131.2705074 | d__Bacteria;p__Proteobacteria;c__Gammaproteobacteria;o__Pseudomonadales;f__Pseudomonadaceae;g__Pseudomonas |
